# Editorial: Modulation of the adaptive immune responses and chronicity of infections with enveloped viruses

**DOI:** 10.3389/fimmu.2023.1137399

**Published:** 2023-02-07

**Authors:** Elias A. Said, Petronela Ancuta, Jean-Pierre Routy, Jean-Pierre Vartanian, Ali A. Al-Jabri

**Affiliations:** ^1^ Department of Microbiology and Immunology, College of Medicine and Health Sciences, Sultan Qaboos University, Muscat, Oman; ^2^ Département de microbiologie, infectiologie et immunologie, Faculté de Médecine, Université de Montréal, Montreal, QC, Canada; ^3^ Centre de Recherche du Centre Hospitalier de l’Université de Montréal (CRCHUM), Montreal, QC, Canada; ^4^ Infectious Disease and Immunity in Global Health Program, Research Institute of McGill University Health Centre, Montreal, QC, Canada; ^5^ Division of Hematology, McGill University Health Centre, Montreal, QC, Canada; ^6^ Virus and Cellular Stress Unit, Department of Virology, Institut Pasteur, Université de Paris Cité, Paris, France

**Keywords:** adaptive immunity, enveloped viruses, HIV, HBV, F-MULV, COVID-19, PRRSV, atherosclerosis

Virus structures began to be understood in the 1930s with Stanley, Bernal, and Fankuchen’s work on the tobacco mosaic virus. Following the initial description, the lipid bilayer membrane on the outer part of the virus differentiated enveloped from non-enveloped viruses. For non-enveloped viruses that do not possess that extra lipid membrane, cell lysis is the most common exit mode from the host cell. Conversely, enveloped viruses contain an outer membrane that surrounds the capsid, and during virus assembly and exit from host cells, these viruses use the host cell membrane itself to assemble their membrane, known as an envelope (1). This process avoids cell lysis and helps enveloped viruses escape the host immune system. Furthermore, the persistence of these viruses, even at low levels, inhibits the development of antigen-independent memory CD8 T cells, resulting in the exhaustion of the immune response with persistent inflammation (2). Enveloped viruses can potentially cause chronic infections that represent major risks to human health. A significant number of enveloped viruses that can cause chronic infections are pandemic, affect hundreds of millions of people, and some of them cause the deaths of millions of humans. This includes some viruses associated with immune deficiencies and cancer, such as Epstein–Barr virus (EBV), hepatitis C and B viruses (HCV and HBV), human cytomegalovirus (HCMV), and human immunodeficiency virus (HIV). The chronic infections caused by these viruses and the lack of efficient vaccines against many of them are important public health challenges. Therefore, to develop efficient treatments and vaccines against these viruses, it is crucial to understand the mechanisms and factors that modulate the adaptive immune responses in chronic infections with enveloped viruses. Additionally, this will help elucidate the criteria, parameters, and correlates that should be considered for the design of new vaccines. This Research Topic contains one review and five research articles that address the mechanism and factors that modulate the adaptive immune responses in chronic infections with enveloped viruses and the consequences on the dynamic of these infections.

HIV infection is a major threat to global health. Potent B cell responses are a key factor in controlling this infection. In their article, Noto et al. investigated the impact of HIV infection on T follicular helper (Tfh) cell subsets and the resulting effect on B cell responses. They reported a decrease in T helper 2 (Th2)-like Tfh cells, which are CXCR3^−^ and produce single interleukin 4 (IL-4) and dual IL-21/IL-4 during HIV replication. The authors also indicated that the percentage of these Th2-like Tfh cells is associated with the total number and cycling of HIV-specific B cells, and that the percentage of Th1-like Tfh cells, which are CXCR3^+^ and produce single interferon-γ (IFN-γ) and dual IL-21/IFN-γ, is associated with HIV-specific B cells that have both CXCR3 and T-bet. IFN-γ induced the expression of CXCR3 and T-bet in B cells, and IL-4 and IL-21 enhanced the maturation of HIV-specific B cells. Additionally, Noto et al. demonstrated that harboring of CXCR3 is associated with a low rate of somatic hypermutations in B cells. Together, this demonstrates that the Th2/Th1-like Tfh imbalance in viremic HIV patients has an impact on B cell responses.

Another interesting phenomena was reported by Podschwadt et al., who demonstrated that the envelope (Env) of the retrovirus Friend murine leukemia virus (F-MuLV) induces IL-10-producing CD4 T cells upon immunization. These CD4 T cells were associated with the suppression of anti-Env CD8 T cell responses. F-MuLV could also reduce the control of tumors by immune cells. This capacity of the Env protein to affect CD8 T cell responses was also observed with gamma retroviruses other than F-MuLV.

Understanding how adaptive immune responses can control the long-term effects of the infection with enveloped viruses is crucial for developing strategies aimed at limiting these infections. Kundura et al. suggested the presence of an association between decreased levels of perforin-expressing CD8 T cells and long coronavirus-induced disease (long COVID) attributed to the persistence of symptoms upon infection with severe acute respiratory syndrome coronavirus-2 (SARS-CoV-2). This sheds light on the importance of boosting antiviral CD8 T cell responses in the early phases of infection with enveloped viruses.

Moreover, strategies aimed at eradicating established chronic infections with enveloped viruses are urgently required. Debelec-Butuner et al. demonstrated the capacity of a combination of bispecific antibodies directed against CD3 or CD28 and the hepatitis B virus (HBV) envelop protein and trispecific antibodies directed against these molecules to engage T cells to eradicate the covalently closed circular DNA of HBV, which is associated with viral chronicity. They compared the capacity of these antibodies to induce cytolytic and cytokine-mediated responses to that of S-CAR T cells. The antibodies induced higher levels of cytokines, and the combined bispecific antibodies induced the highest level of antiviral cytokine production. The study demonstrated the capacity of multispecific antibodies to control viral infections.

The development of treatments that can improve the control of chronic infections with enveloped viruses is a major aspect in managing the spread of these infections. Ruansit et al. demonstrated that molecules in *Hottuynia cordata* (HC), a Chinese herbal remedy, can inhibit infection with porcine reproductive and respiratory syndrome virus (PRRSV). Additionally, HC can induce cytokine production in monocyte-derived macrophages. Treatment with HC resulted in a stronger decrease in the highly pathogenic (HP)-ORRSV-2 load in pigs vaccinated with PRRSV-1 modified-live virus (MLV). This was associated with an increase in IFN regulatory factor 3 (IRF-3). This highlights the potential benefit of combining treatment and vaccination to improve the control of viral infections.

Moreover, in this Research Topic, Talepoor et al. reviewed the literature about the capacity of some chronic viral infections to induce mechanisms that are associated with aging and atherosclerosis, including sustained cytokine signaling and DNA damage. They highlighted the ability of CMV, EBV, and HBV to upregulate senescence-associated molecules, including the tumor-suppressor proteins p16, p21, and p53, and induce replicative senescence and inflammaging. Talepoor et al. also commented on the induction of DNA damage, senescence-associated secretory phenotype (SASP) telomere shortening, and epigenetic modifications of DNA by hepatitis C virus (HCV) and HIV.

Altogether, the authors underlined the importance of several mechanisms involving the adaptive immune system that can control chronic infections with enveloped viruses. This represents a crucial weapon in the arsenal required for the potential prevention and eradication of these viral infections.

## Author contributions

ES wrote the manuscript, AA-J, PA, J-PR and J-PV modified and added parts to the manuscript. All authors contributed to the article and approved the submitted version.
